# Angioedema Related to Angiotensin-Converting Enzyme Inhibitors: Attack Severity, Treatment, and Hospital Admission in a Prospective Multicenter Study

**DOI:** 10.1097/MD.0000000000001939

**Published:** 2015-11-13

**Authors:** Nicolas Javaud, Jallal Achamlal, Paul-George Reuter, Frédéric Lapostolle, Akim Lekouara, Mustapha Youssef, Lilia Hamza, Ahmed Karami, Frédéric Adnet, Olivier Fain

**Affiliations:** Service des Urgences, Centre de Référence associé sur les angiœdèmes à kinines (CRéAk), Assistance Publique-Hôpitaux de Paris, Hôpital Louis Mourier, Université Paris 7, 92 700 Colombes (NJ, JA); Urgences et (P-GR, FL, LH, FA); SAMU-SMUR 93, Assistance Publique - Hôpitaux de Paris, Hôpital Avicenne, Université Paris 13, 93 000 Bobigny (P-GR, FL, FA); Urgences, Assistance Publique - Hôpitaux de Paris, Hôpital Jean Verdier, Université Paris 13, 93 140 Bondy (AL, AK); Urgences, Hôpital d’Eaubonne, 95 600 Eaubonne (AL); Urgences, Hôpital de Gonesse, 95 500 Gonesse (MY); Service de Médecine Interne, DHUi2B, Centre de Référence associé sur les angiœdèmes à kinines (CRéAk), Assistance Publique - Hôpitaux de Paris, Hôpital Saint-Antoine, Université Paris 6, 75 012 Paris, France (OF).

## Abstract

The number of cases of acquired angioedema related to angiotensin converting enzyme inhibitors induced (ACEI-AAE) is on the increase, with a potential concomitant increase in life-threatening attacks of laryngeal edema. Our objective was to determine the main characteristics of ACEI-AAE attacks and, in doing so, the factors associated with likelihood of hospital admission from the emergency department (ED) after a visit for an attack.

A prospective, multicenter, observational study (April 2012–December 2014) was conducted in EDs of 4 French hospitals in collaboration with emergency services (SAMU 93) and a reference center for bradykinin-mediated angioedema. For each patient presenting with an attack, emergency physicians collected demographic and clinical presentation data, treatments, and clinical course. They recorded time intervals from symptom onset to ED arrival and to treatment decision, from ED arrival to specific treatment with plasma-derived C1-inhibitor (C1-INH) or icatibant, and from specific treatment to onset of symptom relief. Attacks requiring hospital admission were compared with those not requiring admission.

Sixty-two eligible patients with ACEI-AAE (56% men, median age 63 years) were included. Symptom relief occurred significantly earlier in patients receiving specific treatment than in untreated patients (0.5 [0.5–1.0] versus 3.9 [2.5–7.0] hours; *P* < 0.0001). Even though icatibant was injected more promptly than plasma-derived C1-INH, there, however, was no significant difference in median time to onset of symptom relief between the 2 drugs (0.5 [0.5–1.3] versus 0.5 [0.4–1.0] hours for C1-INH and icatibant, respectively, *P* = 0.49). Of the 62 patients, 27 (44%) were admitted to hospital from the ED. In multivariate analysis, laryngeal involvement and progressive swelling at ED arrival were independently associated with admission (Odds ratio [95% confidence interval] = 6.2 [1.3–28.2] and 5.9 [1.3–26.5], respectively). A favorable course was observed in all patients. Three patients (5%) experienced a recurrence after angiotensin-converting enzyme inhibitor discontinuation after a median follow-up of 18 (11–30) months.

Two severity criteria—laryngeal edema and the progression of the edema—were independent factors associated with likelihood of hospital admission. Appropriate specific treatments (plasma-derived C1-INH or icatibant) should be available in EDs to prevent possibly life-threatening complications.

## INTRODUCTION

The incidence of acquired angioedema related to angiotensin-converting-enzyme inhibitors (ACEI-AAE) arising during treatment ranges from 0.2% to 0.7% and corresponds to a potentially substantial number of cases.^1–3^ For example, in France, 25% of the 12 million people suffering from high blood pressure and 40% of the 3 million diabetics take angiotensin-converting enzyme inhibitors, and there are 120,000 new heart attack cases every year.^4–6^ In a retrospective observational 5-year study in the United States, ACEI-AAE accounted for 4 of 10,000 visits to the emergency department (ED) each year.^7^

Acquired angioedema related to angiotensin-converting-enzyme inhibitors are characterized by recurrent, localized subcutaneous or submucosal edema. Unlike in hereditary angioedema, the main site of the swelling tends to be the face and not the abdomen.^8,9^ Because of possible upper airway involvement, an attack of ACEI-AAE can be life threatening.^8,10^ Emergency treatment of serious cases requires prompt administration of appropriate specific agents as corticosteroids, antihistamines, and epinephrine have proven ineffective.^9^ Plasma-derived C1-inhibitor (C1-INH) has been used with some success.^11,12^ Recently, a phase 2 comparison with an antihistamine–corticosteroid combination and a 13-case series have highlighted the potential therapeutic efficacy of icatibant.^13,14^

For optimal management, emergency physicians need to be aware of the nature and treatment of ACEI-AAE and of drug (plasma-derived C1-INH and icatibant) availability.^15^ The aim of our prospective observational multicenter study was to determine the main characteristics of ACEI-AAE and, in doing so, the factors associated with hospital admission after a visit to the ED for an attack.

## METHODS

### Design and Setting

This prospective observational study enrolled consecutive patients with an attack of ACEI-AAE who visited one of 4 EDs in north–east Paris between April 2012 and December 2014. The local emergency medical services (EMS) (SAMU 93) had the required logistics capabilities for storage and around the clock delivery of appropriate specific agents (plasma-derived C1-INH and icatibant) to patients or healthcare organizations and for the provision of 24/7/365 expert medical advice over the telephone.^16^ Appropriate care of attacks had been promoted in the EDs by posting placards enhancing EMS awareness of the issue, by providing emergency physicians with instruction sheets on recommended emergency management and prophylaxis of recurrence^17^ and by appointment of 2 contact persons—a member of the reference center for bradykinin-mediated angioedema at the university hospital of Saint-Antoine and a member of the national scientific board of the reference center for bradykinin-mediated angioedema—either of whom could be contacted by the EMS physician in charge if necessary.

Our study complied with Strengthening the Reporting of Observational Studies in Epidemiology guidelines for observational studies.^18^ The protocol was approved by the local ethics committee (*Comité de Protection des Personnes d’Ile de France 10*). According to French legislation, no written informed consent was required. All patients were informed of the study plan. None voiced any opposition.

### Participant Inclusion and Exclusion Criteria

The study inclusion criterion was a diagnosis of angioedema in a patient taking ACEI. The diagnosis was prospectively confirmed by an angioedema specialist on the basis of patient history, a physical examination, the observed inefficacy of antihistamines, corticosteroids, and epinephrine, functional and antigenic C1-INH levels and genetic data. Exclusion criteria were a diagnosis of angioedema other than ACEI-AAE (i.e., hereditary angioedema, acquired angioedema with C1-INH deficiency and histamine-induced angioedema). Patients with a rash, fever, a family history of angioedema or treated with a nonsteroidal anti-inflammatory agent at the time of the visit were also excluded. All patients were seen after the acute attack to perform further laboratory tests, and to establish whether any recurrence had occurred.

### Specific Treatments

Specific treatment for an attack of ACEI-AAE was either plasma-derived C1-INH injection (20 IU/kg solution made up from 2 or more 500 IU-vial) or subcutaneous icatibant injection (30 mg solution in a prefilled syringe) depending upon availability.

### Data Collection and Analysis

For each attack, the emergency physicians prospectively collected the following data on standardized forms: patient sex, age, ACEI in use and when initiated, personal history of angioedema (visits to ED, admissions to intensive care unit, and history of intubation and of tracheotomy), ongoing long-term treatment, possible trigger of attack, day and time of onset of symptoms, day and time of arrival at ED, edema site, edema progression on ED arrival, treatment in ED and when started, course of attack (onset of symptom relief and time of symptom resolution), and decision whether or not to discontinue ACEI. The member of the reference center for bradykinin-mediated angioedema prospectively collected the follow-up data (interval between attacks and follow-up visit data) and any recurrence after ACEI discontinuation. A photographic record of the edema (face and/or tongue) was taken for the medical file after obtaining the patient's consent.

Facial edema was defined as swelling of one or both lips and/or one or both cheeks and/or one or both eyelids. Upper airway involvement was defined as swelling of the tongue and/or larynx (dysphonia and/or dysphagia and/or dyspnea) and/or uvula. Progression of the edema was defined as progressing swelling observed by a physician.

The following times intervals were recorded: time (in hours) before ED arrival (time of symptom onset to time of ED arrival), time (in hours) before decision (time of symptom onset to time of treatment decision), time (in minutes) before therapy (time of ED arrival to hour of plasma-derived C1-INH or icatibant injection), time (in hours) to symptom relief (hour of plasma-derived C1-INH or icatibant injection to hour of onset of symptom relief).

The characteristics of attacks requiring admission to hospital were compared with those not requiring admission.

### Statistical Analysis

Quantitative variables are given as medians with interquartile ranges (IQR) and qualitative data as numbers with percentages. For qualitative variables, differences were tested by the χ^2^ test or, if the validation criteria for this test were not met, by the Fisher exact test. Variables associated with hospital admission from the ED were identified using a logistic regression model. A stepwise multivariate analysis was performed with sex, age, site of attack, and treatment as candidate variables. All tests were 2-sided. A *P* value <0.05 was considered significant. We used R statistical software version 2.15.2 (R Foundation for Statistical Computing, Vienna, Austria).

## RESULTS

### Patient Characteristics

**FIGURE 1:**
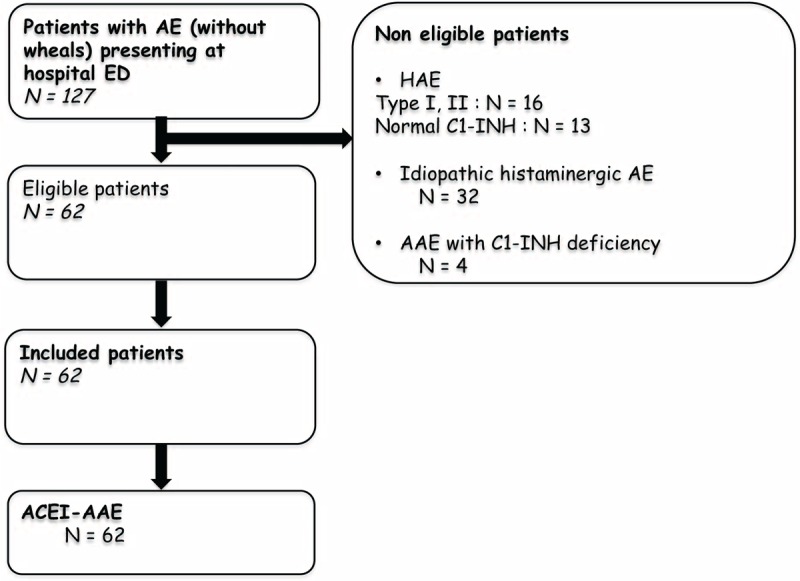
Flowchart of patients. AAE = acquired angioedema, ACEI-AAE = acquired angioedema related to angiotensin-converting enzyme inhibitors, AE = angioedema, HAE = hereditary angioedema.

Overall, 127 patients presented at one of the 4 EDs with an angioedema attack (Fig. 1). Of these 127 patients, 65 were not eligible for the study (29 cases of hereditary angioedema, 32 of histaminergic idiopathic angioedema, and 4 cases of acquired angioedema with C1-INH deficiency). All 62 eligible patients had ACEI-AAE and were included in the study (Table 1). Most were men (56%), median age was 63 years, and 23 (37%) were blacks. The main incriminated ACEI were perindopril (1 in 3 cases), ramipril (1 in 4) and enalapril (1 in 5). The main indication for ACEI treatment was hypertension (3 of 4 cases) (Table 1). In 41 of patients (66%), ACEI-AAE was diagnosed at the first ED visit for an attack. This visit took place a median of 1 year after beginning ACEI treatment. The remaining 21 patients (34%) had experienced a median of 3 attacks over a median of 22 months after treatment introduction and before diagnosis. Three of 8 diabetic patients had received vildagliptine for 8 days, a patient with relapsing prostate cancer had received distilbene for 1 month.

**TABLE 1:**
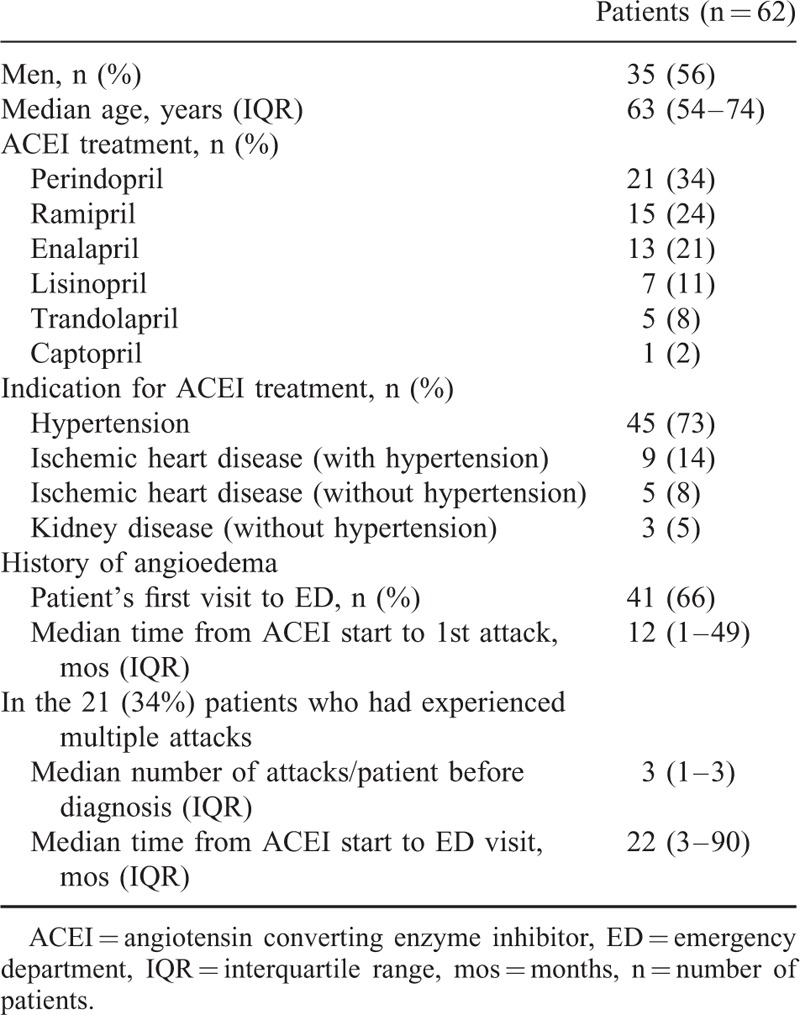
Patient Characteristics

### Angioedema Attacks

**FIGURE 2:**
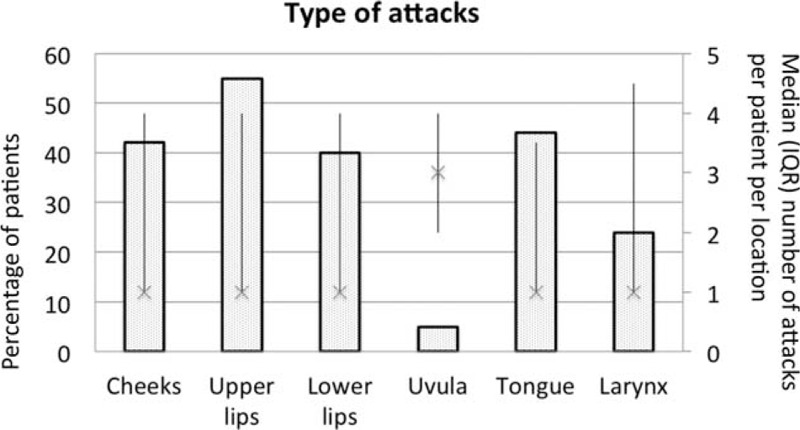
Incidence by edema site and median number of attacks, interquartile ranges, per patient for each Site. Percentage patients: cheeks, upper lip, lower lip, uvula, tongue, larynx. median number of attacks, interquartile ranges, per patient for each site.

The sites of attacks prompting ED visits included at least 1 attack of the upper lip (34/62, 55%), tongue (27/62, 44%), cheeks (26/62, 42%), lower lip (25/62, 40%), larynx (15/62, 24%), or uvula (3/62, 5%; Fig. 1). The upper airways were involved in 30 cases (48%). Fourteen patients (22%) experienced multisite attacks of the head [median 4 (IQR 3–5)] (Fig. 2). Patients arrived at the ED a median of 5.8 (3.1–9.3) hours after the onset of swelling. Median blood pressure at arrival was 145 (130–160)/80 (75–90) mm Hg, median heart rate was 81 (74–90) bpm, arterial oxygen saturation was 98% (97–99), and median body temperature was 36°C (36.4–37.0) and under the lower limit of normal. One patient had inspiratory dyspnea with 86% oxygen saturation on room air, necessitating immediate tracheal intubation. Treatments given were antihistamines to 43 patients (70%), corticosteroids to 35 (56%), and/or epinephrine to 5 (8%) patients. Overall, 41 patients (66%) received either subcutaneous icatibant (30/41 patients, 73%) or intravenous plasma-derived C1-INH (11/41 patients, 27%).

Median time from icatibant or plasma-derived C1-INH injection to onset of symptom relief was significantly shorter in patients receiving plasma-derived C1-INH or icatibant than in patients with no specific treatment (0.5 [0.5–1.0] vs 3.9 [2.5–7.0] hours; *P* < 0.0001). There was no significant difference between the two agents [0.5 (0.5–1.3) hours for plasma-derived C1-INH vs 0.5 (0.4–1.0) hours for icatibant; *P* = 0.49] even though icatibant treatment was initiated much sooner [time from ED arrival to icatibant or plasma-derived C1-INH injection: 1.0 (0.5–1.8) versus 2.0 (1.7–3.0) hours; *P* = 0.02].

### Hospital Admission from Emergency Department

Overall, 27 patients (42%) were admitted to hospital: 22 (82%) in the ED short-stay unit from which they were discharged within 24 hours, 2 (7%) to the intensive care unit, and 3 (11%) in the internal medicine department for a 3-day stay. The course was favorable in all admitted patients. The patient intubated before icatibant injection was successfully extubated on day 2 after the complete resolution of the edema.

Table 2 compares the clinical presentation and treatment of patients who were or were not admitted to hospital from the ED. Rate of hospital admission was significantly higher in cases of laryngeal involvement and progressive swelling, and was less frequent, but not significantly so, in cases of stable or regressive swelling. One admitted patient showed progressive laryngeal swelling despite epinephrine treatment. In multivariate analysis, laryngeal involvement and progressive swelling at ED arrival were independent factors associated with a 6-fold higher likelihood of hospital admission [Odds Ratio (95% CI) = 6.2 (1.3–28.2) and 5.9 (1.3–26.5), respectively] (Table 3).

**TABLE 2:**
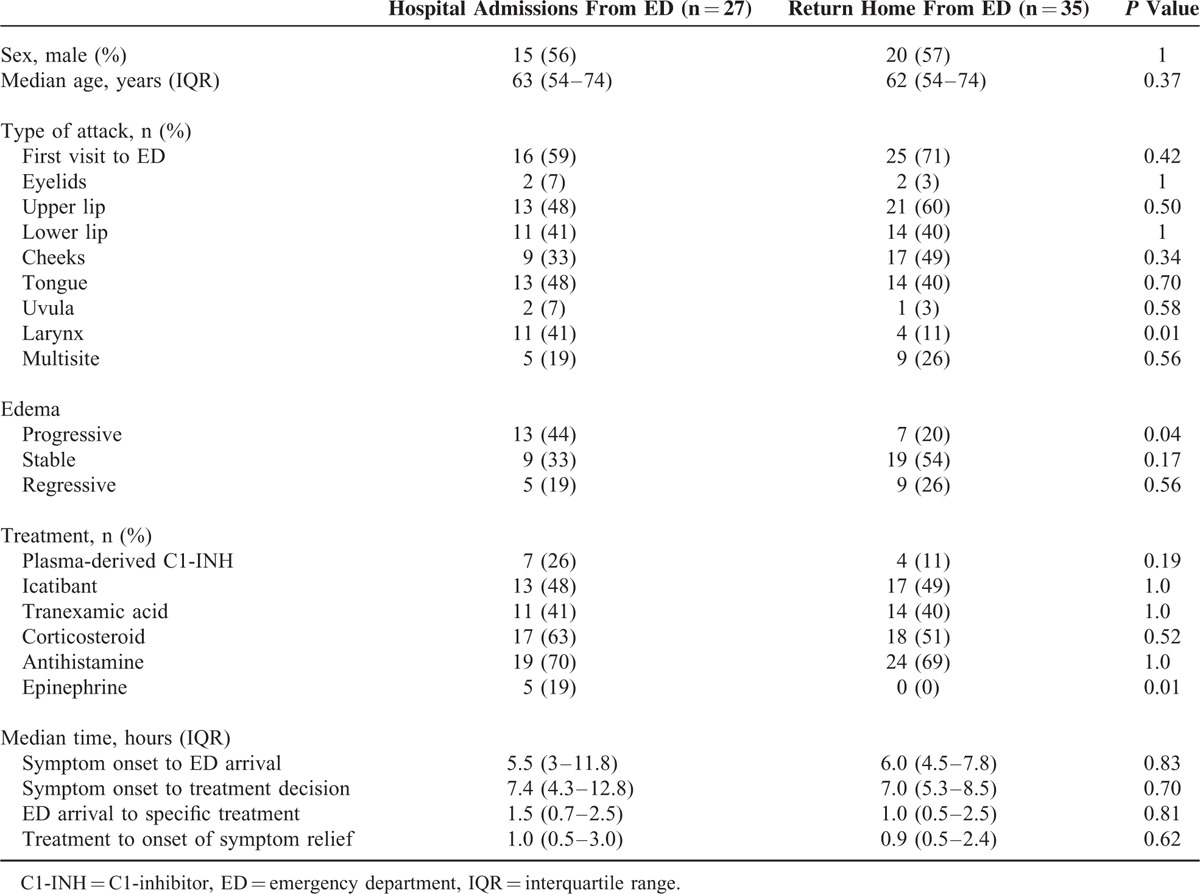
Clinical Presentation and Management

**TABLE 3:**
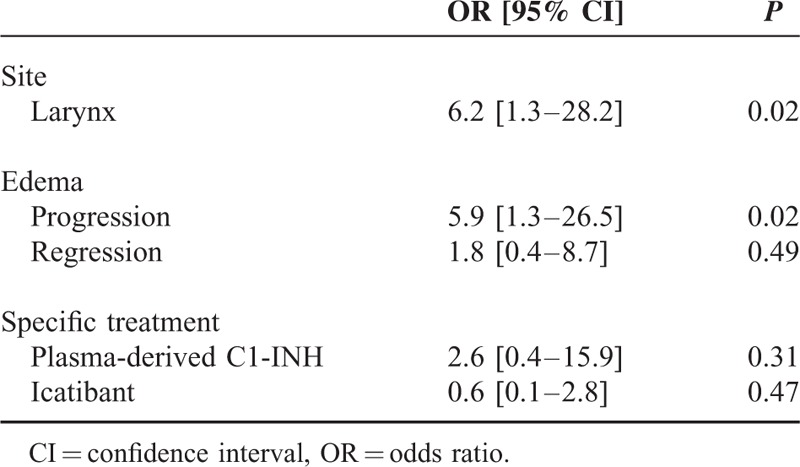
Multivariate Logistic Regression Analysis of Factors Associated With Hospital Admission From the Emergency Department

### Follow-up

Median follow-up was 18 (11–30) months. Angiotensin-converting enzyme inhibitor was discontinued in all patients. Angioedema recurred in 3 female patients (5%) during the first month after ACEI discontinuation. The laboratory work up revealed no quantitative or functional reduction in plasma C1-INH levels.

## DISCUSSION

In the above study of patients with an acute attack of ACEI-AAE, the hospital admission rate from the ED was high (42%) and associated with 2 independent factors, namely, laryngeal involvement (24% incidence) and progressive edema on ED arrival (32% incidence). Two studies have already described an association between laryngeal edema secondary to ACEI treatment and hospital admission from the ED, but they are retrospective.^7,8^ To our knowledge, this is the first analysis of progression of edema on ED arrival in cases of ACEI-AAE.

The 24% incidence of laryngeal involvement in our study was mid of the reported range for ACEI-AAE (2%–59%; Table 4).^2,7,8,13,14,19,20^ No patient died from asphyxia secondary to laryngeal edema and only one patient, in considerable respiratory distress on ED arrival, required immediate intubation before icatibant could be injected. Morbidity/mortality rate from asphyxia is not insignificant but tends to be lower in prospective^13,14^ than retrospective^8,20,21^ studies as patients undergo treatment.

**TABLE 4:**
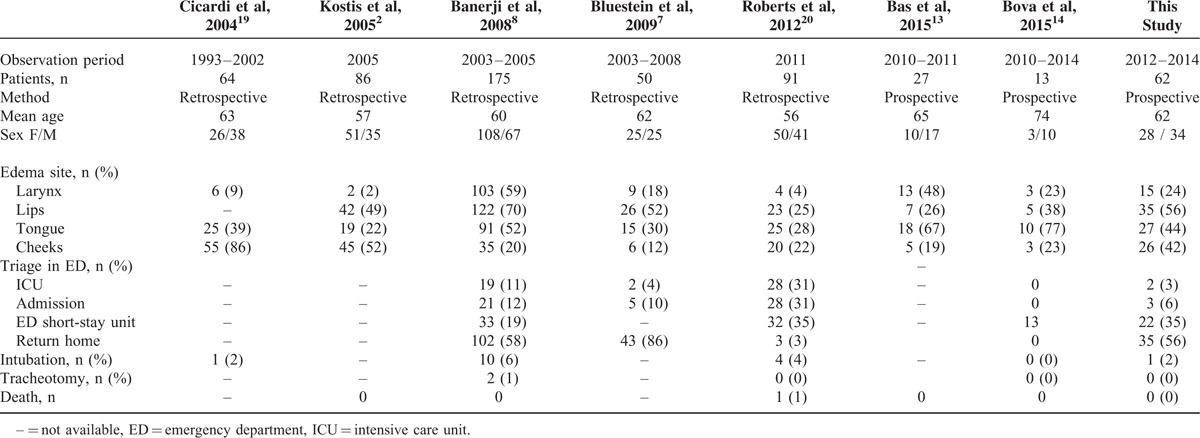
Review of Cases of Acquired Angioedema Related to Angiotensin-Converting Enzyme Inhibitors

For two-thirds of our patients, this was a first angioedema attack prompting an ED visit, confirming the already observed association between a first attack of laryngeal edema because of ACEI and an ED visit.^21^ Two-thirds of patients in the Phase 2 study of icatibant also experienced a first attack of angioedema.^13^

The trigger was identified in 4 patients. Three patients had taken the antidiabetic drug vildagliptin for 8 days. This drug class is associated with an increased risk of angioedema in patients on ACEI [Odds ratio: 4.6 (95% confidence interval 1.6–13.3)] owing to inhibition of a kininase (dipeptidylpeptidase 4 (DDPIV)).^22^ The fourth patient who was suffering from relapsing prostate cancer had recently started taking estrogen, a hormone known to trigger acute attacks in hereditary angioedema.^23^ To our knowledge, this is, however, the first reported case of an estrogen-triggered attack in a patient with ACEI-AAE.

The 42% incidence of macroglossia on ED arrival was close to that reported.^2,7,8,13,14,19,20^ It was higher in patients who were admitted to hospital than in those who were not, but not significantly so. Macroglossia is a common symptom that can cause upper airway obstruction and death, as evidenced by autopsies performed on 7 patients who died from isolated major tongue swelling in the absence of laryngeal edema.^10^

In our study, symptom relief occurred significantly earlier in patients receiving C1-INH concentrate or icatibant than in untreated patients. Prompt treatment of bradykinin-mediated angioedema by plasma derived C1-INH or icatibant remains a challenge for EMS.^24^ Our result underscores the potential efficacy of these drugs in ACEI-AAE and confirms the recently reported efficacy of icatibant in a phase 2 study^13^ and the efficacy of plasma-derived C1-INH in several clinical case reports.^11,12^ Plasma-derived C1-INH decreases bradykinin production. Other bradykinin-degrading enzymes (aminopeptidase P and carboxypeptidase N) thus have a better chance to work.^25^ There was, however, no significant difference in median time to onset of symptom relief between the 2 drugs even though icatibant was injected significantly more promptly than plasma-derived C1-INH. Icatibant is injected subcutaneously using a prefilled syringe; plasma-derived C1-INH solution has to be made up before being administered intravenously. The efficacy observed in our study was particularly noteworthy as most patients suffered from extensive and progressive edema on ED arrival, at a potentially life-threatening site (48% upper airway cases), and had failed to respond to antihistamine, corticosteroid, or epinephrine before being administered plasma-derived C1-INH or icatibant.

Angiotensin-converting enzyme inhibitor discontinuation prevented recurrence in all but 3 of the 62 patients. A high recurrence rate is unlikely because of the long-median follow-up (18 months) after discontinuation. This is in contrast to the 50% recurrence rate observed in an earlier study.^26^ Nevertheless, a laboratory work up and monitoring during 6 to 12 months are necessary to rule out diagnoses, such as hereditary angioedema and histamine-induced or noninduced idiopathic angioedema.^17^

## STUDY LIMITATIONS

A strong point of our study is the prospective standardized collection of events leading up to hospital admission. The data were easy to collect and provided a precise clinical description of attacks, treatments, and management times. Results, however, cannot be generalized to all EDs as the 4 participating EDs were particularly well informed in ACEI-AAE management and the cases they were called upon to treat were probably more serious than those generally encountered. The main limitation was the small number of patients even though the study is the largest observational prospective study carried out over a relatively short time scale so far. Another limitation is the lack of a control group.

## CONCLUSIONS

Emergency physician knowledge and awareness of ACEI-AAE is a key factor in patient management. Attacks of laryngeal edema and the progression of the edema were risk factors associated with hospital admission from the ED. Despite the severity of the cases, response to treatment with the specific agents plasma-derived C1-INH and icatibant was favorable. Extensive or progressive edema, in particular, might be a sign that prompt emergency treatment by these agents is required. To prevent cases of asphyxiation, plasma-derived C1-INH, and/or icatibant should be available in all EDs.
